# Fault-controlled natural hydrogen reservoirs as underground hydrogen storage sites

**DOI:** 10.1073/pnas.2617910123

**Published:** 2026-09-28

**Authors:** Lei Hou, Zhengxin Zhang, Derek Elsworth, Fengshou Zhang, Samintha Perera, Qinghua Lei, Mengke An, Lei Zhang, Qi Zhou

**Affiliations:** ^a^ China-UK Low Carbon College, Shanghai Jiao Tong University, Shanghai 201306, China; ^b^Departments of Energy and Mineral Engineering and Geosciences, Earth and Mineral Sciences Energy Institute, and Center for Geomechanics, Geofluids, and Geohazards, Pennsylvania State University, University Park, PA 16802; ^c^ Department of Geotechnical Engineering, College of Civil Engineering, Tongji University, Shanghai 200092, China; ^d^ Department of Infrastructure Engineering, Faculty of Engineering, The University of Melbourne, Melbourne, VIC 3010, Australia; ^e^ Department of Earth Sciences, Uppsala University, Uppsala 75105, Sweden; ^f^ Department of Civil and Environmental Engineering, The Hong Kong Polytechnic University, Hong Kong 100872, China; ^g^Research Center of Multiphase Flow in Porous Media, School of Petroleum Engineering, China University of Petroleum (East China), Qingdao 266580, China

**Keywords:** underground hydrogen storage, natural hydrogen, fault reactivation, hydrogen loss, storage capacity

## Abstract

Underground hydrogen storage requires geological formations with demonstrated hydrogen containment, yet no conventional storage candidate (salt caverns, aquifers, or depleted hydrocarbon reservoirs) offers direct, prior evidence of H_2_-specific sealing. We propose that subcommercial natural hydrogen reservoirs, where H_2_ has accumulated over geological timescales, can serve as precertified storage sites. This proposition is evaluated using the Bulqizë fault-controlled natural H_2_ reservoir in Albania. We develop a coupled geofluid–geomechanical model from exploration data incorporating depth-dependent temperature and permeability gradients across the 5-km vertical extent of the fault zone, hydrogen dissolution under two end-member hydrogeological configurations, and fault stability assessed through slip tendency analysis. Our results show that hydrogen losses from dissolution and abiotic consumption remain below 2% of total inventory under conservative upper-bound assumptions—independently consistent with the sustained natural H_2_ accumulation observed in the reservoir. We identify two distinct, permeability-dependent fault reactivation modes: localized instability near the injection interval at low permeabilities, and instability at the fault–caprock interface at high permeabilities. Parametric analysis across 72 combinations of injection rate, location, and permeability defines safe operating envelopes and yields optimized storage capacities of 3.5 to 4.8 × 10^8^ m^3^. A 10-y cyclic injection–production simulation demonstrates stable throughput of 7.5 × 10^5^ m^3^·d^−1^ with progressively decreasing fault reactivation risk—a counterintuitive finding driven by pressure homogenization through gas redistribution. These results establish natural hydrogen reservoirs as a viable class of underground storage sites.

## Significance

Large-scale underground hydrogen storage is essential to support a hydrogen-based energy economy, yet identifying geological sites that are both technically and economically viable remains contentious. We demonstrate that natural hydrogen reservoirs—where hydrogen has accumulated over geological time—offer a fundamentally different site-selection pathway. Using exploration data from the Bulqizë reservoir in Albania (with one of the largest world-recorded H_2_ flow rates), we show that the host fault remains stable over a decade of cyclic injection-production, and that the hydrogen losses are below 2%. These findings establish natural hydrogen reservoirs as a category of underground storage sites, potentially repurposing hundreds of known natural hydrogen occurrences worldwide—particularly those below the commercial threshold—as high-value storage candidates.

Underground hydrogen storage (UHS) is considered a prerequisite for integrating hydrogen into the global energy system, providing the large-scale, long-duration buffering capacity that surface storage cannot deliver (1–4). In contrast to surface storage technologies—where advances in materials science have rapidly diversified options (high-pressure tanks, cryogenic systems, metal hydrides, etc.)—UHS faces a fundamental scale gap between labs and fields, which slows down the scalable UHS process. Governing subsurface hydrogen behaviors, including dissolution, adsorption, microbial consumption, and geochemical reactions, have been extensively studied at the laboratory scale (5–7), yet extrapolating these findings to heterogeneous reservoir conditions spanning kilometers remains deeply uncertain. Bridging this gap requires field-scale pilot projects involving geological surveys, well drilling, and long-term monitoring—efforts so resource-intensive that only a handful of national-level programs have been undertaken (e.g., Underground Sun Storage in Austria and HyPSTER in France) (8, 9). As a result, the number of geologically validated storage concepts is limited.

Among the few options explored, salt caverns are the most technically mature, yet their deployment is constrained by stringent geological prerequisites—requiring thick, homogeneous halite formations, and by the high cost of solution mining for cavern creation. Once operational, caverns remain susceptible to creep-induced volume loss and demand continuous pressure management to maintain structural integrity (10, 11). Porous media reservoirs (saline aquifers and depleted oil and gas fields) offer substantially greater storage capacity, but the increased flow resistance inherent in rock pores tends to limit the overall hydrogen recovery rate. More critically, the dispersion of hydrogen within rock pores vastly expands the interfacial contact area with the formation, potentially exacerbating hydrogen losses via aqueous dissolution, microbial consumption, and redox reactions. These consumptive processes frequently liberate impurity gases such as CO_2_ and H_2_S, thereby contaminating the stored hydrogen—a challenge further exacerbated by the residual gases in depleted reservoirs (5, 12, 13). Consequently, extant field trials for hydrogen storage in porous media have predominantly employed impure hydrogen streams (enriched with N_2_, CH_4_, and CO_2_) rather than high-purity hydrogen (14, 15). In all cases, the ability of a given formation to retain hydrogen over operational timescales has yet to be demonstrated by direct geological evidence—a gap that costly, short-duration pilot tests can only partially address.

Recent explorations of natural hydrogen (H_2_) reservoirs offer a fundamentally different approach to closing this evidence gap. Hundreds of natural H_2_ occurrences have been documented worldwide (16), yet the commercial outlook for direct extraction remains sobering: observed flow rates typically fall between 10^5^ and 10^7^ m^3^·y^−1^, at least an order of magnitude below the threshold for economically viable production (17, 18), and most future discoveries are expected to remain subcommercial. We propose that these subcommercial reservoirs should be reconsidered not as failed exploration targets, but as precertified UHS sites. The existence of a natural H_2_ accumulation constitutes direct geological evidence that H_2_ consumption rates have remained below deep supply rates over geological timescales, which provides the containment guarantee that conventional UHS candidates lack. Moreover, continuous deep H_2_ replenishment driven by serpentinization or radiolysis serves as a natural cushion gas that offsets storage losses—unlike engineered cushion gases (e.g., CO_2_, N_2_) that introduce impurities and incur additional costs. Meanwhile, coupling storage infrastructure with ongoing natural H_2_ exploration amortizes costs across both functions. However, natural hydrogen retention under undisturbed geological conditions does not guarantee containment under the repeated injection–production cycles, which must be demonstrated through process-based analysis.

Here we evaluate this proposition using the Bulqizë natural H_2_ reservoir in Albania—one of the most extensively characterized natural hydrogen systems to date (19). The reservoir is hosted within a large fault zone in Jurassic ophiolites, steeply dipping at 80°, with a vertical extent of up to 5 km and temperatures spanning 40 to 160 °C. Gas samples from the mine yield H_2_ concentrations of 84% by volume, with an estimated annual release of at least 200 tonnes—one of the highest recorded natural H_2_ flow rates worldwide (19). Several attributes make this reservoir a particularly informative test case for UHS: elevated temperatures and the limited availability of CO_2_ and other carbon substrates suppress microbial and abiotic hydrogen consumption; gas-filled structures with no degassing observed in the upper mine or Batra deposit (indicating effective long-term sealing); and relatively high fault-zone permeability favors rapid injection and extraction. At the same time, the fault-controlled geometry and vertically extensive reservoir column present one of the most geomechanically challenging scenarios for hydrogen storage, making Bulqizë a stringent end-member test of feasibility.

Evaluating UHS feasibility in such reservoirs poses modeling challenges absent from conventional storage settings. The Bulqizë fault zone extends to 5,000 m depth, producing steep gradients in temperature, permeability, and in situ stress that uniform-property models cannot capture. Formation water introduces dissolution losses whose magnitude depends on poorly constrained hydrogeological conditions, while clumped-isotope evidence for abiotic methane generation indicates that reactive hydrogen consumption must be quantified at storage timescales (19). Most critically, the low viscosity of H_2_ promotes rapid pressure transmission along fracture networks, raising the risk of injection-induced fault reactivation (20–22). No prior study has integrated these geofluid and geomechanical processes for a natural hydrogen reservoir.

Here we develop a coupled geofluid–geomechanical model incorporating depth-dependent property gradients, hydrogen dissolution under two hydrogeological configurations, and fault stability evaluated through slip tendency (ST) analysis. Our results show that hydrogen losses remain below 2% of total inventory under conservative assumptions—independently consistent with the geological evidence of sustained natural H_2_ accumulation. We identify two distinct, permeability-dependent fault reactivation modes and demonstrate that, under optimized injection strategies, storage capacities of 3.5 to 4.8 × 10^8^ m^3^ are achievable. A 10-y cyclic simulation reveals that repeated injection–production progressively stabilizes the fault—a counterintuitive finding driven by pressure homogenization through gas redistribution—confirming the viability of natural H_2_ reservoirs for decadal-scale UHS. These findings point to a broader opportunity: repurposing the growing inventory of subcommercial natural hydrogen discoveries as certified storage sites for the hydrogen economy.

## Results

### Hydrogen Losses in the Bulqizë Reservoir Are Inherently Constrained.

Potential hydrogen losses arise from dissolution into formation water (a largely reversible process) and from microbial or abiotic consumption (irreversible pathways). We quantify each under conservative upper-bound assumptions to establish whether hydrogen losses constitute a first-order barrier to storage.

#### Dissolution in connected bottom water.

**Fig. 1.**
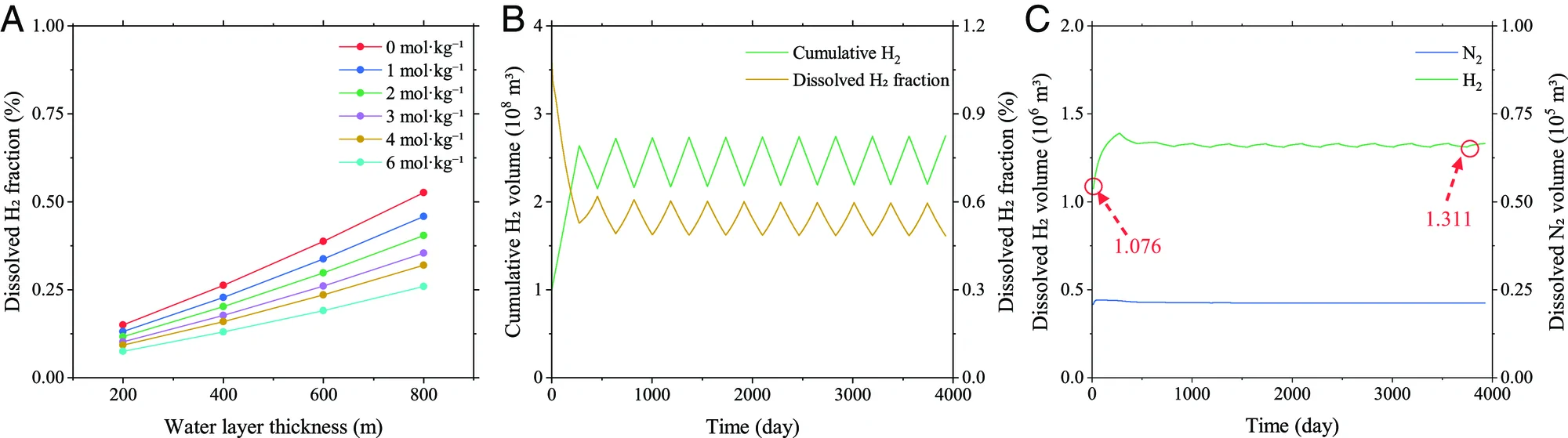
Simulation of H_2_ dissolution under a connected bottom-water configuration. (*A*) Dissolved H_2_ fraction vs. bottom-water thickness at different salinities; (*B*) Cumulative H_2_ volume and dissolved H_2_ fraction over 10-y cyclic operation; (*C*) Dissolved H_2_ and N_2_ volumes in bottom water over 10-y cyclic operation.

In the Bulqizë reservoir, formation water from the ore zone (Level 20) is characterized by Na–Cl type chemistry and elevated salinity. Increasing salinity raises the Henry constant of H_2_ and suppresses its solubility (*SI Appendix*, Fig. S1); we therefore adopt pure water to define an upper bound on dissolution. Owing to strong structural control on fluid flow and heterogeneous water availability, we first model dissolution under a connected bottom-water configuration, in which a mobile aqueous phase accumulates within the highly transmissive fault system. As only H_2_ and N_2_ are present as free gas (CH_4_ being largely abiotic and associated with water-limited conditions), dissolution of these two species in bottom water is considered. Hydrogen was injected into Layer 41 at 6 × 10^5^ m^3^·d^−1^ for 9 mo. Under bottom-water thicknesses of 200, 400, 600, and 800 m, the maximum dissolved hydrogen fraction reaches only 0.53% of the total stored volume, even in the most extreme case (800 m water column, pure water; Fig. 1*A*).

To capture dissolution losses at the engineering scale, a 10-y cyclic injection–production simulation was conducted. Hydrogen was injected into Layer 41 (depth of 4,433 m) and produced from Layer 21 (depth of 2,433 m) with annual cycles and a working gas rate of 3 × 10^5^ m^3^·d^−1^. Owing to the vertical separation between the injection interval and the bottom-water zone, dissolution exhibits a delayed response: total hydrogen inventory increases immediately upon injection, whereas dissolved hydrogen rises more gradually, causing the dissolved fraction to initially decrease before increasing after a time lag (Fig. 1*B*). The volume of dissolved hydrogen increases from 1.08 × 10^6^ to 1.31 × 10^6^ m^3^ over 10 cycles—a net increment of only 2.35 × 10^5^ m^3^. Variations between successive cycles are small and diminish progressively, indicating that dissolution-related losses during cyclic operation tend toward a quasi-steady state (Fig. 1*C*).

#### Dissolution in bound water.

**Fig. 2.**
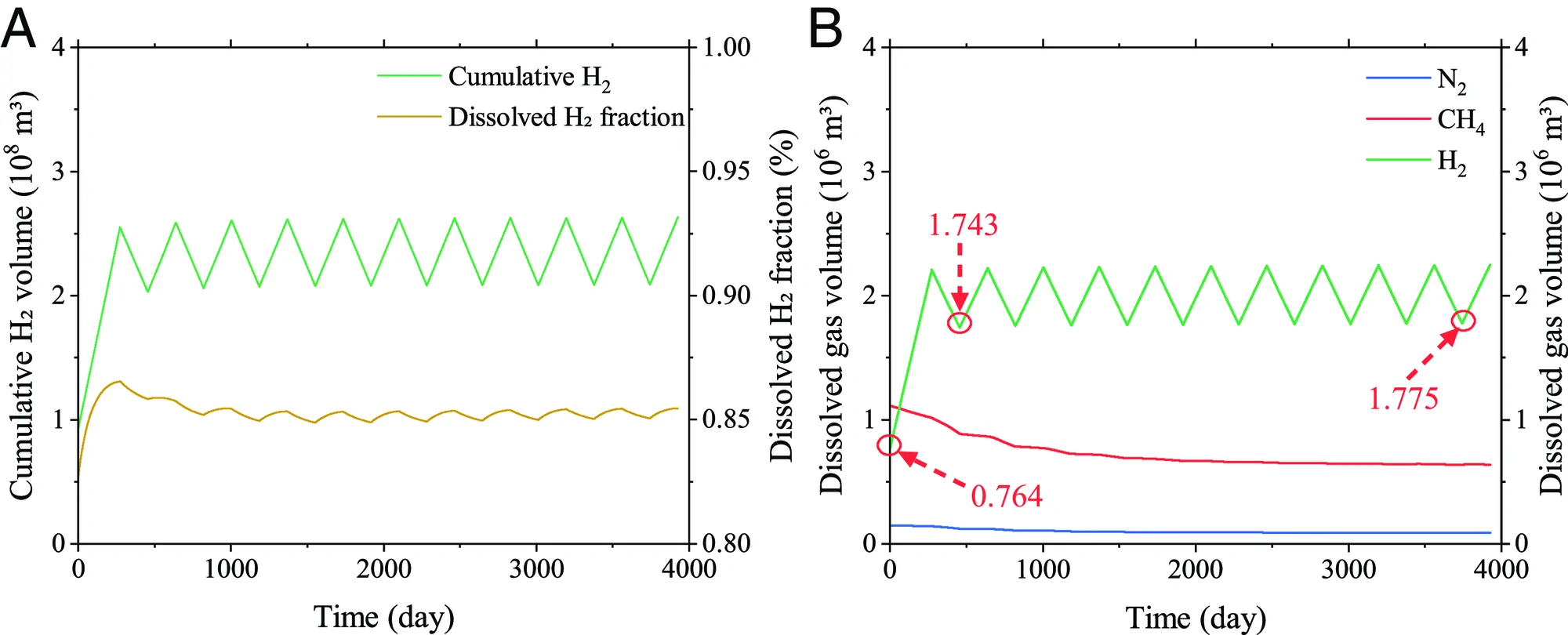
Simulation of H_2_ dissolution under a bound-water configuration. (*A*) Cumulative H_2_ volume and dissolved H_2_ fraction over 10-y cyclic operation in the bound-water system; (*B*) Dissolved H_2_, CH_4_, and N_2_ volumes in formation water over 10-y cyclic operation.

For the second end-member configuration—a water-limited state in which serpentinization outpaces fluid replenishment, leaving water primarily as bound or residual saturation—we model dissolution under saturations of 5, 10, 15, and 20%, using the same injection scheme. Across all four cases, dissolved hydrogen remains within the same order of magnitude, reaching a maximum of 2.21 × 10^6^ m^3^ (0.87% of total stored volume) after 9 mo of injection at 20% water saturation, using a reservoir-wide H_2_ Henry constant corresponding to 160 °C and zero salinity (Fig. 2*A*). Based on this conservative case, a 10-y cyclic simulation using a multiwell scheme at 3 × 10^5^ m^3^·d^−1^ confirms that dissolution is largely reversible: dissolved hydrogen increases during injection and exsolves during production, stabilizing around a quasi-steady dissolved fraction (Fig. 2*A*). The volume of dissolved hydrogen in formation water increases from 0.76 × 10^6^ to 1.78 × 10^6^ m^3^ over 10 cycles, but the difference between the first and eleventh injection cycles is only 3.2 × 10^4^ m^3^ (Fig. 2*B*). Across both hydrogeological configurations, dissolution-related losses remain on the order of 1% of the total hydrogen inventory (~10^8^ m^3^).

#### Microbial consumption.

For the four principal metabolic pathways for subsurface hydrogen consumption—methanogenesis, sulfate reduction, acetogenesis, and iron reduction—the reported optimal temperature ranges collectively span 0 to 40 °C, reaching only the lower bound of the Bulqizë reservoir (~40 °C), with critical thresholds ranging from 72 to 122 °C (*SI Appendix*, Table S1). Consistent with this thermal constraint, CH_4_ isotopic analyses from the reservoir indicate an abiotic rather than microbial origin for methane, and gas samples contain neither water nor oxidized carbon species (CO_2_, CO), further disfavoring active microbial metabolism during storage (19).

#### Abiotic consumption.

Despite the suppression of microbial activity, abiotic hydrogen consumption via CO_2_ hydrogenation (CO_2_ + 4H_2_ → CH_4_ + 2H_2_O) is thermodynamically feasible and must be evaluated. Although CO_2_ was not detected in the sampled gas phase, oxidized carbon may persist in dissolved form within highly alkaline formation waters. We estimate an upper bound on the available carbon by considering the measured alkalinity of the ore-zone water (pH 11.1, salinity 17.8 mM, OH^−^ concentration ~1.3 mM), yielding dissolved inorganic carbon bounds of 8.25 to 16.5 mM. Because high water availability inhibits abiotic CO_2_ hydrogenation, carbon supplied by bottom water is expected to contribute only marginally, whereas relatively dry gas–rock domains in the middle and upper reservoir are more favorable reaction sites. Assuming 20% bound-water saturation (total bound-water mass of 4.67 × 10^8^ kg), the stoichiometry of CO_2_ hydrogenation constrains maximum hydrogen consumption to 1.5 to 3.1 × 10^7^ mol at the reservoir scale—less than 0.3% of the total hydrogen inventory (~10^10^ mol). Abiotic consumption is thus carbon-limited rather than H_2_-limited in this ultramafic system. We note that this assessment is based on simplified assumptions regarding carbon supply; additional carbon sources cannot be ruled out and warrant investigation in future studies.

#### Independent validation from natural accumulation.

The simulation-derived upper bounds on hydrogen losses are independently corroborated by the geological record. The Bulqizë reservoir retains an estimated 5,000 to 50,000 tonnes of in-place H_2_ despite continuous exposure to all loss mechanisms over geological timescales, releasing at least 200 t·y^−1^ with no observed depletion trend (19). This sustained accumulation provides a model-independent integral constraint: the time-averaged net hydrogen flux (deep supply minus total losses) must have remained positive throughout the reservoir’s history. Our process-based simulations independently confirm this condition, with all quantified loss pathways (1% of H_2_ loss from dissolution, 0.3% of H_2_ loss from abiotic consumption, and even less microbial consumption) summing to well under 2% of the stored inventory. The convergence of forward modeling and geological evidence on the same conclusion—that hydrogen losses do not constitute a first-order barrier to storage—provides a level of assurance unavailable for any conventional UHS site.

### Fault Permeability Controls Two Distinct Reactivation Modes.

Although hydrogen losses are limited from a risk-assessment perspective, the fault-controlled structure of the Bulqizë reservoir introduces a separate risk: injection-induced fault reactivation. We systematically investigate how fault permeability and injection location govern reactivation behavior, using the coupled geofluid–geomechanical model with fault stability evaluated through maximum ST.

#### Permeability-dependent reactivation mechanisms.

**Fig. 3.**
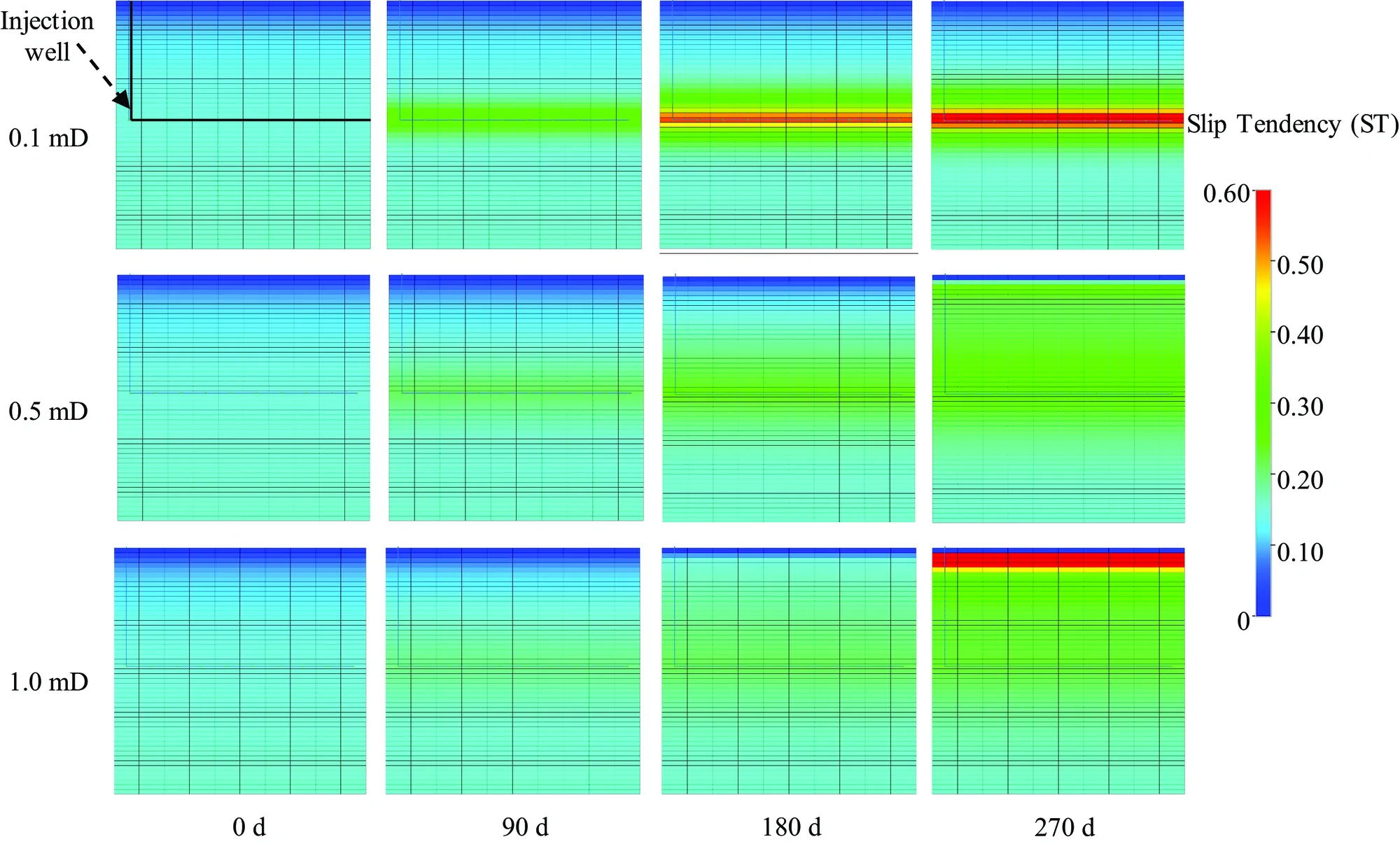
Spatiotemporal evolution of fault ST under different fault-bottom permeabilities. Rows indicate fault-bottom permeabilities of 0.1, 0.5, and 1.0 mD, and columns indicate injection times of 0, 90, 180, and 270 d. Colors denote the magnitude of fault ST.

Hydrogen was injected via a horizontal well at the mid-width of the fault (Layer 25, depth 2,833 m) at a constant rate of 5 × 10^5^ m^3^·d^−1^ for 9 mo, under three fault-bottom permeabilities (0.1, 0.5, and 1.0 mD). The simulations reveal two fundamentally distinct reactivation modes depending on permeability (Fig. 3). At low permeability (0.1 mD), fault ST is greatest near the injection well and spreads gradually to the surrounding volume, driven by local pressure buildup within the poorly transmissive formation under elevated crustal stress at depth. At high permeability (1.0 mD), the dominant risk shifts to the fault–caprock interface: hydrogen migrates rapidly upward along the permeable fault, accumulating beneath the seal and triggering a sharp increase in ST once a critical pressure threshold is reached. Reactivation at this location occurs abruptly, in contrast to the gradual evolution observed at low permeability. At intermediate permeability (0.5 mD), both mechanisms operate simultaneously, producing a characteristic “double-peak” pattern in which elevated ST develops both near the injection interval and at the fault–caprock junction. These results demonstrate that fault permeability exerts a first-order control on not only the magnitude but also the location and timing of reactivation risk—information essential for designing safe injection strategies.

#### Parametric analysis of injection location.

**Fig. 4.**
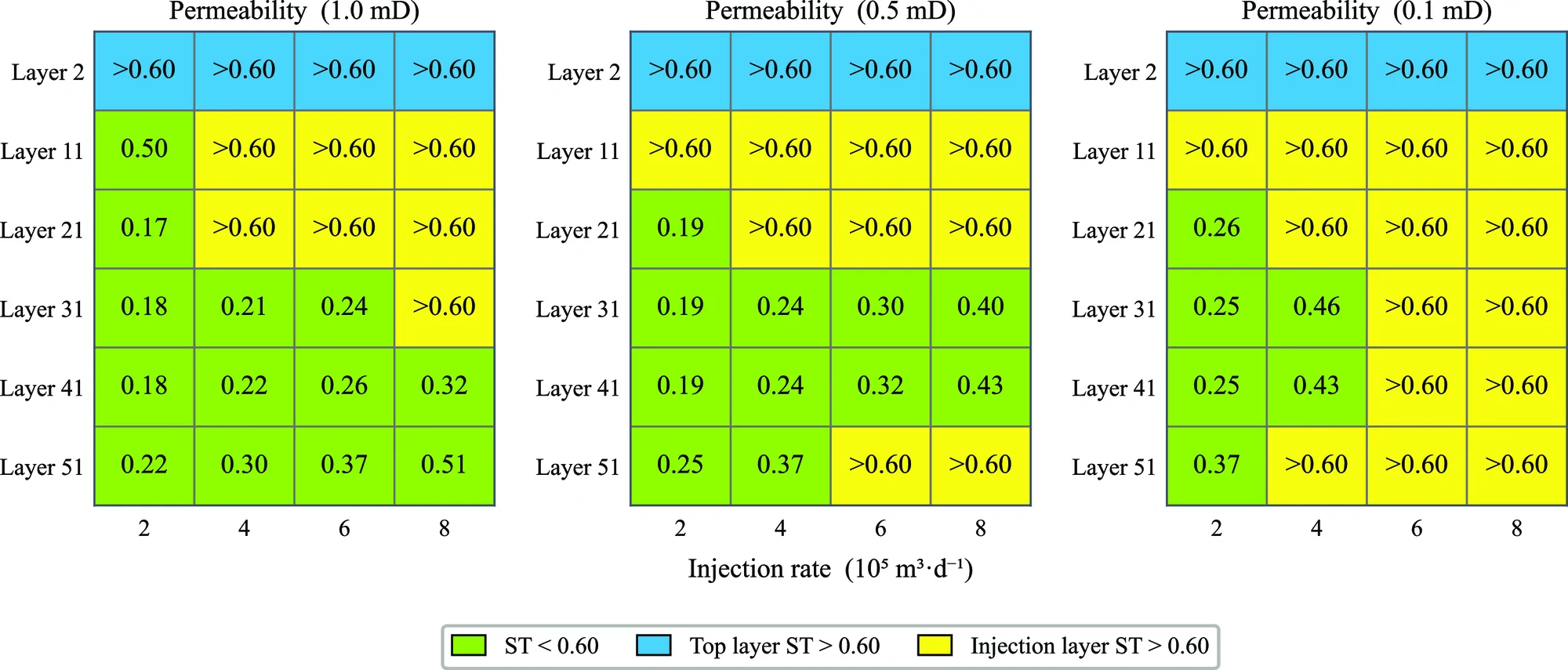
Maximum ST matrix for H_2_ injection. Values show the maximum ST for each injection condition. Green indicates ST < 0.60, blue indicates top-layer reactivation (ST > 0.60), and yellow indicates injection-layer reactivation (ST > 0.60).

The significant vertical extent of the Bulqizë reservoir (up to 5,000 m) makes conventional injection at the reservoir base potentially unsuitable due to extreme depth. We therefore map the full parameter space of injection location, rate, and permeability by simulating six injection layer positions (grid layers 2, 11, 21, 31, 41, and 51, corresponding to depths of 533 to 5,433 m), four injection rates (2 × 10^5^ to 8 × 10^5^ m^3^·d^−1^), and three permeabilities (0.1, 0.5, and 1.0 mD), yielding 72 parameter combinations (Fig. 4).

#### The results reveal systematic patterns.

Injection at the fault–caprock interface (Layer 2, 533 m) triggers reactivation under all parameter combinations regardless of rate or permeability, rendering it universally unsuitable. For shallow-to-mid-depth injection (Layers 11 and 21, 1,433 m and 2,433 m), reactivation can be avoided only at the lowest injection rate (2 × 10^5^ m^3^·d^−1^), yielding insufficient storage volumes. Injection at the reservoir base (Layer 51, 5,433 m) causes local H_2_ accumulation in the low-permeability deep formation, elevating pressure and triggering instability even at moderate rates. Layer 41 (depth 4,433 m) emerges as the optimal injection position: it avoids reactivation under most rate–permeability combinations, maintaining ST below the critical threshold of 0.6 across the widest operating envelope. At intermediate permeability (0.5 mD), Layer 41 sustains safe injection up to 8 × 10^5^ m^3^·d^−1^, providing the best balance between storage volume and geomechanical safety.

These parametric results underscore a key insight: in fault-controlled natural hydrogen reservoirs, neither the deepest nor the shallowest injection points are optimal. Instead, an intermediate depth that balances pressure dissipation against upward migration risk provides the safest operating window—a finding that generalizes beyond the Bulqizë case to other fault-hosted storage candidates.

### Cyclic Operations Stabilize Fault Behavior Over Decadal Timescales.

Building on the parametric analysis above, we optimize injection strategies to maximize H_2_ storage capacity (reported as total gas volumes) while maintaining fault stability, and then evaluate long-term performance through a decade of cyclic injection and production.

#### Injection strategy optimization.

**Fig. 5.**
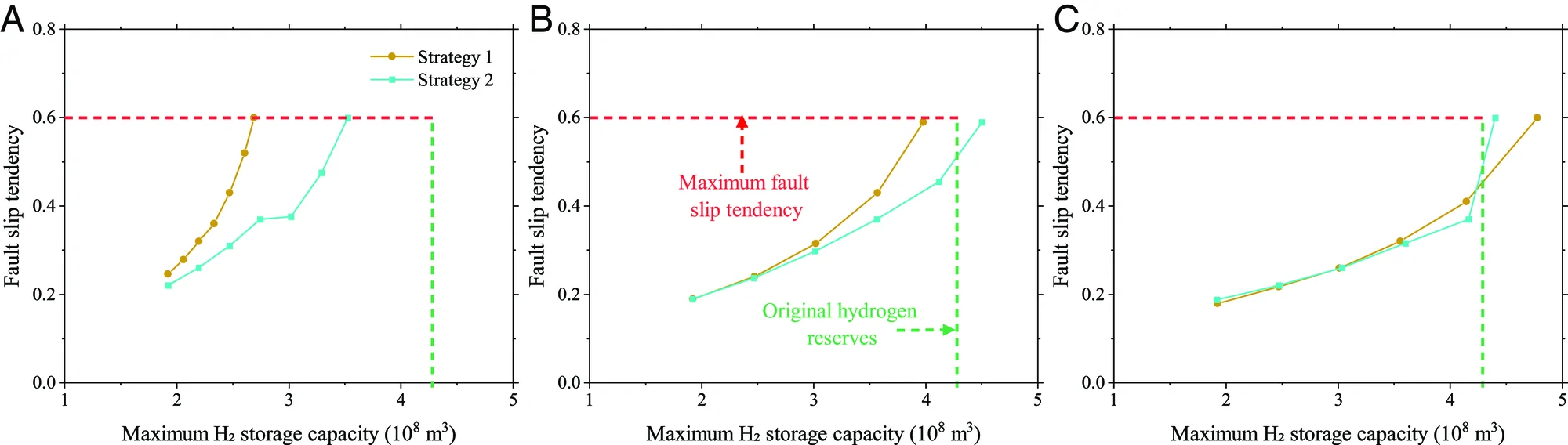
Effects of two injection strategies on fault ST under different permeability conditions: (*A*) k = 0.1 mD; (*B*) k = 0.5 mD; and (*C*) k = 1.0 mD.

We compare two strategies under a 9-mo injection phase at a constant rate across three permeabilities (0.1, 0.5, and 1.0 mD). Strategy 1 employs a single injection layer (Layer 41), with parameters optimized to avoid reactivation. Strategy 2 distributes injection equally among three layers (Layers 31, 41, and 51), maintaining the same total injection volume—motivated by the observation that at low permeabilities, the sweep coverage of a single well is limited (Fig. 5 *A*–*C*).

The two strategies show permeability-dependent trade-offs. At high permeability (1.0 mD), both strategies yield similar reactivation risk at lower injection volumes, but as the injected volume approaches the original natural H_2_ capacity of the fault (4.27 × 10^8^ m^3^), risk rises sharply—more so for Strategy 2, because the inclusion of Layer 31 introduces reactivation at the fault–caprock interface. At low to intermediate permeabilities (0.1 and 0.5 mD), however, Strategy 2 demonstrates a clear advantage: the distributed injection produces lower maximum ST and achieves higher storage capacity at the same risk level.

Under optimized conditions, the maximum H_2_ storage capacities for Strategy 1 are 4.78 × 10^8^ m^3^ (1.0 mD), 3.98 × 10^8^ m^3^ (0.5 mD), and 2.69 × 10^8^ m^3^ (0.1 mD). For Strategy 2, the corresponding values are 4.40 × 10^8^, 4.50 × 10^8^, and 3.53 × 10^8^ m^3^. Notably, both strategies can exceed the estimated original natural H_2_ reserves at their respective optimal permeabilities—Strategy 1 at 1.0 mD and Strategy 2 at 0.5 mD—confirming that properly designed injection can fully utilize the reservoir’s storage potential. Trend analysis further reveals that fault reactivation risk increases gradually during early injection but accelerates sharply as the injected volume approaches the storage limit, underscoring the importance of maintaining injection rates within a low, stable risk envelope.

#### 10-y cyclic injection and production.

Based on the above analysis, we select Strategy 2 at 0.5 mD for long-term performance evaluation. An initial injection of 1 × 10^6^ m^3^·d^−1^ over 9 mo establishes a total gas volume of 4.12 × 10^8^ m^3^, closely matching the original reservoir capacity. Thereafter, annual cycles comprise an injection phase (April–September) and an extraction phase (October–March) at a throughput of 7.5 × 10^5^ m^3^·d^−1^, with extraction wells placed in Layers 21, 31, and 41 (spaced 1,000 m apart) to optimize sweep efficiency under the prevailing permeability and pressure conditions.

**Fig. 6.**
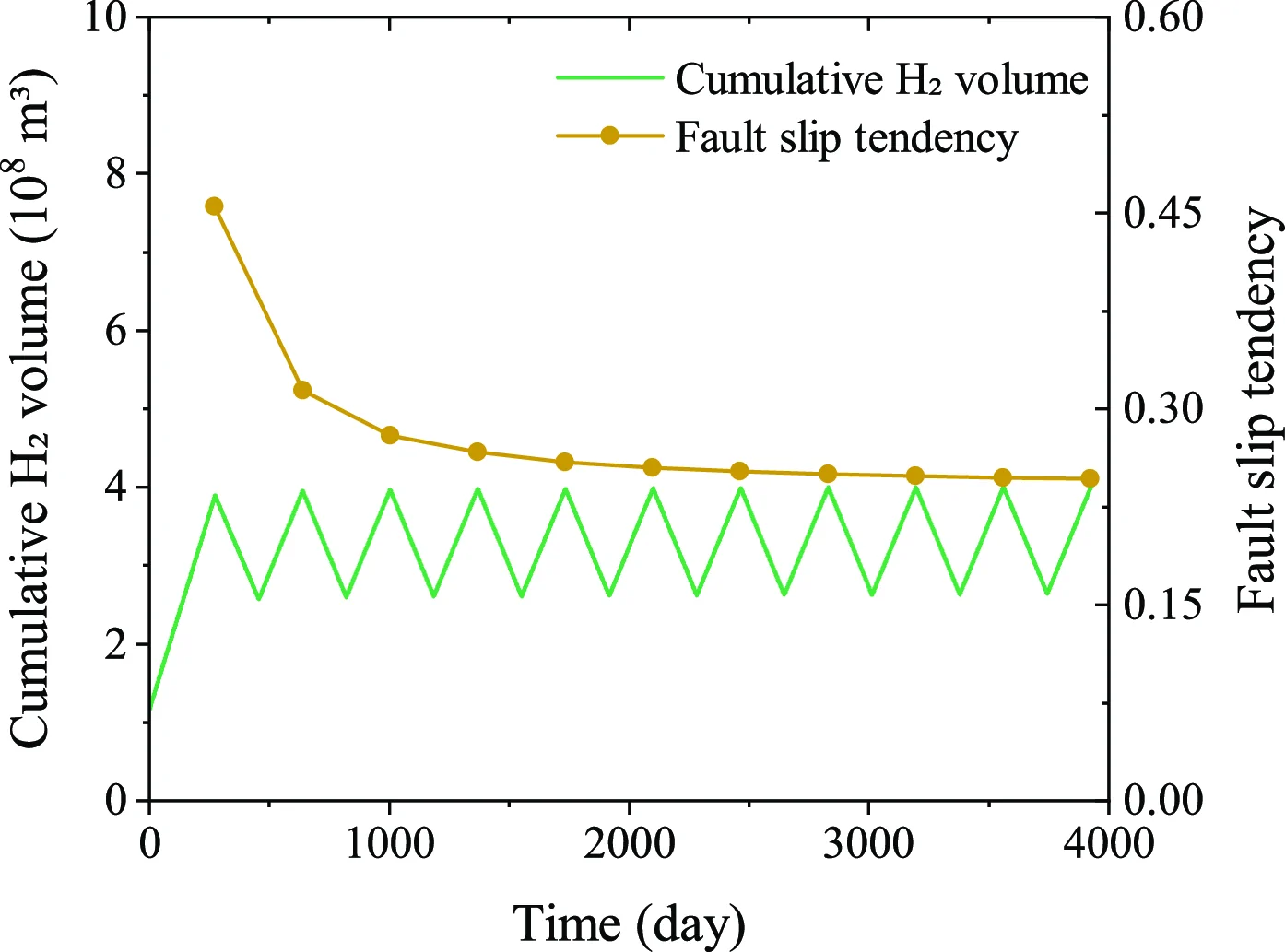
Cumulative H_2_ and fault ST over a decade of cyclic injection and production.

The simulation demonstrates that the reservoir can sustain a stable throughput of 7.5 × 10^5^ m^3^·d^−1^ over the full 10-y period (Fig. 6). The total gas extracted per cycle is approximately 1.37 × 10^8^ m^3^, representing 33.25% of the total storage capacity. Most significantly, the maximum fault ST during the injection phase decreases progressively with each successive cycle (Fig. 6). This counterintuitive result—that repeated injection–production stabilizes rather than destabilizes the fault—reflects the progressive homogenization of pressure distribution within the fault zone through repeated gas redistribution. As H_2_ migrates and redistributes over multiple cycles, localized pressure concentrations that initially drove elevated ST are gradually dissipated, yielding a more uniform stress state. This finding carries an important implication: the risk profile of hydrogen storage in fault-controlled natural reservoirs improves with operational maturity, in contrast to conventional concerns that cyclic loading progressively degrades fault integrity.

## Discussion

### Natural Hydrogen Reservoirs as a Distinct Class of Storage Sites.

Our results demonstrate that hydrogen losses in the Bulqizë reservoir remain below 2% of total inventory and that optimized injection strategies can achieve storage capacities exceeding the estimated natural H_2_ reserves, with fault reactivation risk decreasing over successive operational cycles. These findings position natural hydrogen reservoirs as a functionally distinct category within the UHS portfolio, with characteristics that differ qualitatively from established options. Salt caverns offer high recovery rates but suffer from relatively constrained storage capacities and geomechanical instabilities (23, 24). Depleted gas reservoirs provide large capacity and existing infrastructure, though residual hydrocarbon contamination and H_2_-specific sealing remain concerns (25, 26). Natural hydrogen reservoirs offer compensating advantages unavailable elsewhere: geological self-certification of H_2_ containment over millennia and continuous deep hydrogen replenishment (a cost-free cushion gas). Rather than competing for rapid-turnaround applications, natural hydrogen reservoirs can serve as a strategic complement to salt caverns and depleted fields, trading cyclic agility for superior purity, long-term integrity, and large-scale seasonal buffering.

### Broader Relevance of the Bimodal Fault Reactivation Mechanism.

Our simulation reveals that fault permeability governs two distinct reactivation modes—localized instability near the injection interval at low permeabilities vs. instability at the fault–caprock interface at high permeabilities. This carries implications beyond hydrogen storage. Fluid injection into fault zones is central to multiple subsurface energy operations, including hydraulic fracturing (27), CO_2_ geological sequestration (28), wastewater disposal (29), and enhanced geothermal systems (30). In each case, the conventional focus has been on reactivation near the injection point, where pressure perturbation is greatest. Our results show that in high-permeability fault systems, the critical failure location can be spatially decoupled from the injection point, migrating to structural discontinuities (such as the fault–caprock junction) where stress contrasts concentrate. This spatial decoupling implies that monitoring strategies focused solely on the injection interval may miss the most critical reactivation zone, representing a particularly challenging scenario that demands integrated, full-fault monitoring approaches. These insights may inform risk assessment protocols across the broader spectrum of subsurface injection activities.

### Limitations.

Several modeling simplifications warrant acknowledgment. The sequential coupling approach used here (flow→geomechanics) does not capture the full feedback between stress-induced permeability changes and subsequent fluid redistribution; fully coupled hydro-mechanical simulations would refine quantitative predictions, particularly near the reactivation threshold. The grid resolution (5,100 cells across a reservoir with a 5-km vertical extent) provides adequate representation of first-order processes but may not resolve fine-scale pressure gradients near the injection well or fault–caprock interface. Rock fatigue under repeated pressure cycling, a known mechanism of progressive failure in geological materials (31), is not incorporated and could modify long-term fault behavior beyond the 10-y horizon simulated here. Finally, our hydrogen loss estimates rely on upper-bound assumptions for carbon availability and water distribution; as exploration of the Bulqizë system advances, refined geochemical and hydrogeological constraints will enable tighter quantification of these processes.

### From Bulqizë to a Global Opportunity.

The Bulqizë reservoir represents one of the most geomechanically challenging end-members for UHS—a fault-controlled, vertically extensive, steeply dipping system where injection-induced reactivation is a primary risk. That even this demanding scenario yields favorable storage performance suggests that natural hydrogen reservoirs with more benign geological characteristics may offer greater storage potential with reduced geomechanical complexity, for instance, intracratonic basin settings with sedimentary caprocks (e.g., Bourakebougou, Mali), and shallower ophiolite-hosted systems with lower thermal gradients. The global inventory of documented natural H_2_ occurrences continues to expand (16), yet the commercial outlook for standalone hydrogen extraction remains constrained by insufficient flow rates for the vast majority of these sites (17, 18). Our findings offer a pathway to unlock the value of these subcommercial discoveries. By repurposing them as UHS sites, the exploration infrastructure, geological data, and well assets that would otherwise represent sunk costs become enabling investments for storage deployment. This coupling of natural hydrogen exploration with engineered storage creates a synergistic model that simultaneously advances resource delineation and expands the number of certified storage sites—a convergence with significant implications for hydrogen economy roadmaps worldwide.

## Materials and Methods

A three-dimensional numerical model of the Bulqizë fault-controlled natural H_2_ reservoir was constructed using the CMG-GEM compositional reservoir simulator (32), based on exploration data from the Bulqizë ophiolite region (19). The model comprises a chromite-bed matrix (volume 2.75 km^3^) and an embedded fault-bounded reservoir (volume 5.0 × 10^−2^ km^3^), discretized into 5,100 cells (10 × 10 × 51) with local grid refinement within the fault zone. The reservoir is tilted at 80° with a 15° cell inclination, sealed by no-flow lateral boundaries. Depleted initial conditions are imposed (initial pressure 8 MPa) to represent a storage-ready interval. Deep H_2_ supply is set to zero, as low-temperature serpentinization yields only ~10^5^ mol·y^−1^—negligible on the timescale of this study.

Temperature and permeability vary with depth to capture the nonstationary conditions across the 5-km vertical extent of the reservoir. Surface temperature is 25 °C with a geothermal gradient of 25 °C·km^−1^. Permeability decays exponentially with depth following $k\;=\;k_{i}\exp\left[-\gamma \left(p-p_{i}\right)\right]$, where $k_{i}$ and $p_{i}$ are the reference permeability and initial pressure at the first grid layer, respectively, p is the initial pressure at the corresponding layer, and γ is the permeability compressibility (0.044 MPa^−1^ for the fault zone). Fluid properties are computed via the Peng-Robinson equation of state, with gas viscosity determined from the Jossi-Stiel-Thodos correlations. Full model parameters are provided in *SI Appendix*, Tables S2 and S3.

Hydrogen dissolution in formation water is modeled using Henry’s law with pressure-, temperature-, and salinity-dependent Henry constants. Two end-member hydrogeological configurations are considered: i) a connected bottom-water zone at the base of the fault, and ii) distributed bound water at residual saturations of 5 to 20% throughout the reservoir.

Fault reactivation is evaluated by sequentially coupling fluid flow with a geomechanical model (CMG-Geomechanics module). The in situ stress field assumes a rock density of 3,000 kg·m^−3^, a vertical stress gradient of 29.43 kPa·m^−1^, and a horizontal-to-vertical stress ratio of 0.6. Fault constitutive behavior follows a slip-weakening model in which the friction coefficient decreases from a maximum of 0.7 to a residual value of 0.3 over a critical slip displacement of 0.15 m, based on serpentinite friction experiments (33). Fault stability is assessed through the maximum slip tendency $ST\;=\;\frac{\tau_{\max}}{C\;+\;\sigma_{n}^{\prime }\;\times \;SF}$, where $\tau_{max}$ is the maximum shear stress, *C* is cohesion and $\sigma_{n}^{\prime }$ is the effective normal stress, and *SF* is the slip friction coefficient. Reactivation is considered to occur when *ST* exceeds 0.6, a threshold derived from seismic monitoring studies (34, 35). Complete geomechanical parameters and governing equations are provided in *SI Appendix*.

ChatGPT (OpenAI, GPT-5.5 Thinking) was used for grammar checking, language polishing, and improving clarity and readability during manuscript preparation. It was not used to generate original research ideas, data analysis, figures, images, results, or conclusions. All AI-assisted edits were carefully reviewed, fact-checked, and approved by the authors, who take full responsibility for the final content of the manuscript.

## Supplementary Material

Appendix 01 (PDF)

## Data Availability

The source data supporting Figs. 1–6 and *SI Appendix*, Fig. S1 are available in Zenodo at https://doi.org/10.5281/zenodo.22290002 (36). All other study data are included in the article and/or *SI Appendix*.
