# Supplementary material for: Fault-controlled natural hydrogen reservoirs as underground hydrogen storage sites
Source: Proc Natl Acad Sci U S A. 2026 Sep 28;123(41):e2617910123. doi: 10.1073/pnas.2617910123 (PMC13663760; doi:10.1073/pnas.2617910123)
Supplement: Supplementary file 1 [file pnas.2617910123.sapp.pdf]

Supporting Information for
Fault-controlled natural hydrogen reservoirs as underground hydrogen storage sites

Lei Hou [a*], Zhengxin Zhang [a*], Derek Elsworth [b], Fengshou Zhang [c*], Samintha Perera [d], Qinghua Lei [e], Mengke An [f], Lei Zhang [g], Qi Zhou [a]

[a] China-UK Low Carbon College, Shanghai Jiao Tong University, Shanghai 201306, China
[b] Departments of Energy and Mineral Engineering and Geosciences, Earth and Mineral Sciences Energy Institute, and Center for Geomechanics, Geofluids, and Geohazards, Pennsylvania State University, University Park, PA 16802, USA
[c] Department of Geotechnical Engineering, College of Civil Engineering, Tongji University, Shanghai 200092, China
[d] Department of Infrastructure Engineering, Faculty of Engineering, The University of Melbourne, Melbourne, VIC 3010, Australia
[e] Department of Earth Sciences, Uppsala University, Uppsala 75105, Sweden
[f] Department of Civil and Environmental Engineering, The Hong Kong Polytechnic University, Hong Kong 100872, China
[g] Research Center of Multiphase Flow in Porous Media, School of Petroleum Engineering, China University of Petroleum (East China), Qingdao 266580, China

* Lei Hou, Zhengxin Zhang, and Fengshou Zhang.
Email: leihou.upc@gmail.com (L. Hou), zhangzhengxin99@sjtu.edu.cn (Z. Zhang), fengshou.zhang@tongji.edu.cn (F. Zhang).

This PDF file includes:

Supporting text
Figure S1
Tables S1 to S3
SI References

Supporting Information Text

Methods

Geological context for Bulqizë H_2 reservoir.
The Bulqizë reservoir is hosted in the Jurassic ultramafic massif of Albania. The massif covers about 370 km^2, extends to a depth of up to 6 km, and contains folded and faulted concordant chromite ore bodies within the mantle sequence. The lithological assemblage includes harzburgites with dunite lenses and cumulates including dunites, pyroxenites, and gabbros.

Rock samples collected from the deeper mine levels include massive chromitites, serpentinized dunites, and serpentinized harzburgites from the ore body, mineralized zone, host rock, and fault zone. The H_2-rich tectonic zone is about 10 m wide, strikes NNW–SSE, and dips westward at about 80°. Structural observations at levels L17 and L19 show multiple fracture families, and the fault planes are commonly serpentinized, filled with calcite veins, and marked by slickensides.

Previous interpretation of the Bulqizë system suggests that H_2 is stored within fractures and connected porosity of the fault zone, which acts as a porous reservoir. The estimated fault-zone volume ranges from 5.0×10^{-3} to 5.0×10^{-2} km^3, and an equivalent porosity of 5% was assumed for this highly damaged zone. Because the available Bulqizë data do not report site-specific permeability measurements for the full fault-zone reservoir, the permeability contrasts used in this study were selected with reference to published permeability ranges for fault-related rocks, and are used as sensitivity cases to represent uncertainty in the hydraulic behavior of the fault-controlled reservoir (1).

Geomechanical model of the Bulqizë H_2 reservoir.
First, the fault slip displacement is calculated using a slip-weakening model to describe the relationship between the slip friction coefficient and slip displacement, as expressed in Eq. 1 (2):

$$D_s = \frac{\tau_{max}}{K_t} \tag{1}$$

where D_s is the fault slip displacement, τ_{max} is the maximum shear stress and K_t is the fault tangential stiffness.

The relationship between fault slip displacement D_s and critical slip displacement D_{cri} is used to select the corresponding slip friction coefficient SF. When the slip displacement exceeds the critical value $D_s \geq D_{cri}$, the slip friction coefficient is set to the minimum friction coefficient $fric_{min}$; if the slip displacement is less than the critical value $D_s < D_{cri}$, the slip friction coefficient varies with slip displacement, as calculated using Eq. 2 (3):

$$SF = fric_{max} - \frac{(fric_{max} - fric_{min})}{D_{cri}} D_s \tag{2}$$

where SF is the slip friction coefficient, $fric_{max}$ is the maximum slip friction coefficient, $fric_{min}$ is the minimum slip friction coefficient and D_{cri} is the critical slip displacement.

The parameters here are derived from fault reactivation experiments on serpentinite (4). The experiments show that the maximum slip friction coefficient is 0.7, which decreases to 0.3 when the slip displacement reaches the critical value of 0.15 m and remains constant as the slip displacement increases. If the friction coefficient changes, the friction angle also changes. In this case, it is referred to as the active friction angle, which causes the fault to reactivate earlier than when the sliding friction is constant. The fault slip tendency may be evaluated using Eq. 3 (5):

$$ST = \frac{\tau_{\max}}{C + \sigma_n^{'} \times SF} \tag{3}$$

where ST is the fault slip tendency, C is the cohesion, and $\sigma_n^{'}$ is the normal effective stress acting across the fault. Seismic monitoring studies have shown that when the fault slip tendency exceeds 0.6, the fault may reactivate, potentially triggering an earthquake (e.g., the 1995 Hyogo-

ken Nanbu earthquake, with an estimated slip tendency of around 0.6, which was selected as the threshold for maximum slip tendency (6, 7)). Therefore, in this study, the maximum slip tendency ST_{max} is set to 0.6. If the calculated slip tendency exceeds this value $\mathrm{ST} > ST_{max}$, fault reactivation will occur.

Figures

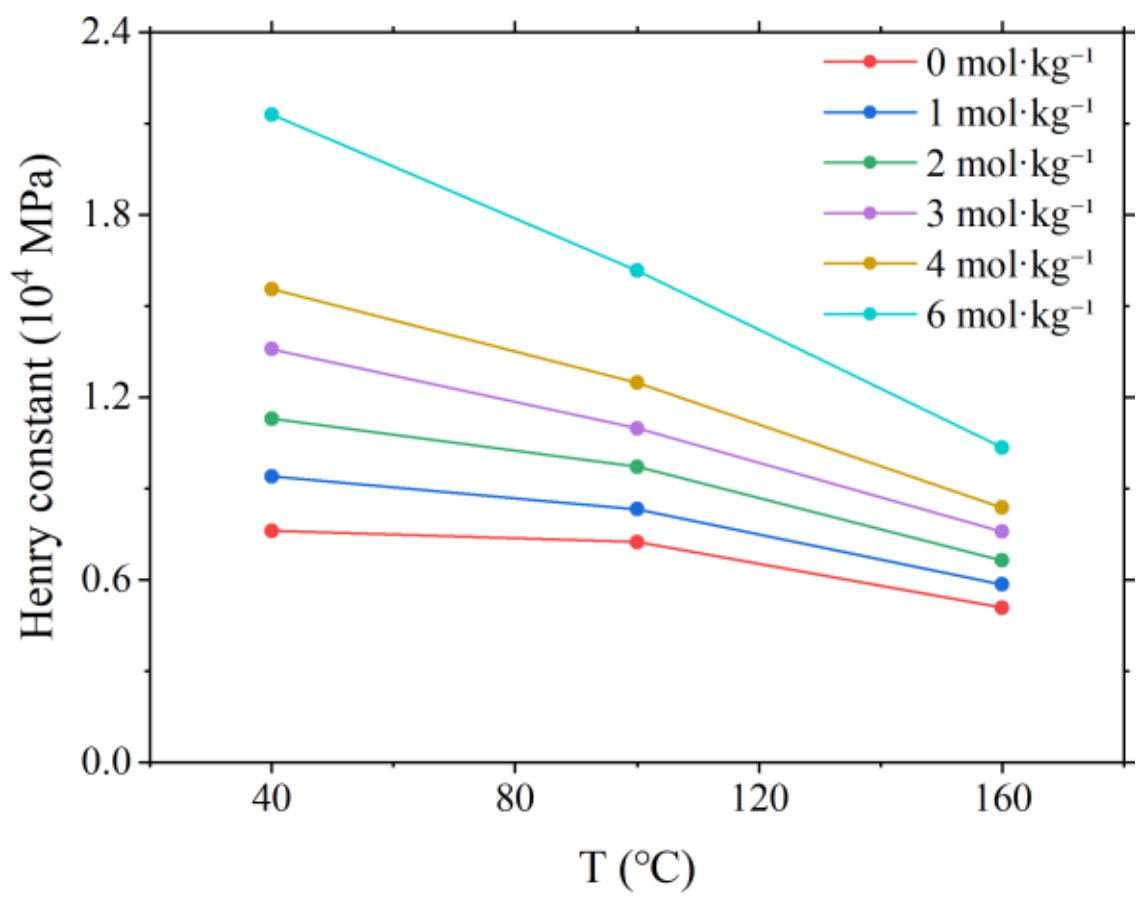

Fig. S1. H_2 Henry constant as a function of temperature at different salinities.

Tables

Table S1. Key parameters of microbial hydrogen consumption (8).

Class of microorganism	Reaction	H_2 consumption rate (nM hour^{-1})			Temperature (°C)	
		Lab	Field	Wells	Optimal condition	Critical condition
Methanogens	$1/4HCO_3^- + H_2 + 1/4H^+ \rightarrow 1/4CH_4 + 3/4H_2O$	$0.008\sim5.8\times10^5$	~1185	~4533	30~40	122
Sulfate reducers	$1/4SO_4^{2-} + H_2 + 1/4H^+ \rightarrow 1/4HS^- + H_2O$	$0.005\sim130\times10^5$	0.05~ 351	~2544	20~30	113
Homoacetogens	$1/2HCO_3^- + H_2 + 1/4H^+ \rightarrow 1/4CH_3COO^- + H_2O$	$0.2\sim5.0\times10^5$	/	/	20~30	72
Iron(III) reducing bacteria	$2FeOOH + H_2 + 4H^+ \rightarrow 2Fe^{2+} + 4H_2O$	$0.005\sim2.2\times10^5$	/	/	0~30	90

Table S2. Parameters representing geological and fluid models (1, 9, 10).

Parameters	Matrix	Fault	Unit
Volume	2.75	5.0×10^{-2}	km^3
Length	1000	1000	m
Width	500	10	m
Height	5500	5000	m
Top depth	450	/	m
Reservoir dip angle	80	80	°
Grid-cell inclination	15	15	°
Porosity	2	5	%
Bottom permeability	1.0×10^{-7}	0.1-1	mD
Pressure	30	8	MPa
Gas viscosity	0.01	0.01	cP
Permeability compressibility	0	0.044	MPa^{-1}
Pore volume compressibility	1.45×10^{-4}	4.35×10^{-4}	MPa^{-1}

Table S3. Parameters defining geomechanical model (4, 6, 7, 9).

Parameters	Matrix	Fault	Unit
Young's modulus	3×10^4	3×10^4	MPa
Poisson's ratio	0.2	0.2	-
Rock density	3000	3000	kg/m^3
Cohesion	/	3	MPa
Maximum fault friction	/	0.7	-
Maximum fault slip tendency	/	0.6	-
Fault cohesion	/	1×10^{-2}	MPa
Fault tangential stiffness	/	1×10^3	MPa/m
Minimum fault friction	/	0.3	-
Critical slip displacement	/	0.15	m

SI References

1. Evans JP, Forster CB, & Goddard JV (1997) Permeability of fault-related rocks, and implications for hydraulic structure of fault zones. *Journal of Structural Geology* 19(11):1393-1404.
2. Andrews DJ (1976) Rupture velocity of plane strain shear cracks. *Journal of Geophysical Research* 81(32):5679-5687.
3. Aagaard BT, Heaton TH, & Hall JF (2001) Dynamic earthquake ruptures in the presence of lithostatic normal stresses: Implications for friction models and heat production. *Bulletin of the Seismological Society of America* 91(6):1765-1796.
4. Proctor BP*, et al.* (2014) Dynamic weakening of serpentinite gouges and bare surfaces at seismic slip rates. *Journal of Geophysical Research: Solid Earth* 119(11):8107-8131.
5. Kano Y*, et al.* (2014) Analysis of changes in stress state and fault stability related to planned CO2 injection at the Tomakomai offshore site. *Energy Procedia* 63:2870-2878.
6. Sekiguchi H, Irikura K, & Iwata T (2000) Fault geometry at the rupture termination of the 1995 Hyogo-ken Nanbu earthquake. *Bulletin of the Seismological Society of America* 90(1):117-133.
7. Yukutake Y, Takeda T, & Yoshida A (2015) The applicability of frictional reactivation theory to active faults in Japan based on slip tendency analysis. *Earth and Planetary Science Letters* 411:188-198.
8. Hou L*, et al.* (2026) Natural hydrogen migration and accumulation as a blueprint for enhanced underground hydrogen storage. *Renewable and Sustainable Energy Reviews* 237:117050.
9. Truche L*, et al.* (2024) A deep reservoir for hydrogen drives intense degassing in the Bulqizë ophiolite. *Science* 383(6683):618-621.
10. Zhang Z, Sun G, Zhou X, Dang K, & Su X (2023) Dynamic Fracture Characterization Using Multiphase Rate Transient Analysis of Flowback and Production Data. in *SPE/IATMI Asia Pacific Oil & Gas Conference and Exhibition* (Society of Petroleum Engineers, Jakarta, Indonesia).
